# Multiscale Microstructures and Microstructural Effects on the Reliability of Microbumps in Three-Dimensional Integration

**DOI:** 10.3390/ma6104707

**Published:** 2013-10-22

**Authors:** Zhiheng Huang, Hua Xiong, Zhiyong Wu, Paul Conway, Frank Altmann

**Affiliations:** 1 School of Physics and Engineering, Sun Yat-sen University, 135 West Xingang Road, Guangzhou 510275, China; E-Mails: xh4mg@hotmail.com (H.X.); wuzhiy2@mail2.sysu.edu.cn (Z.W.); 2 School of Mechanical and Manufacturing Engineering, Loughborough University, Loughborough LE11 3TU, UK; E-Mail: p.p.conway@lboro.ac.uk; 3 Fraunhofer Center for Applied Microstructure Diagnostics CAM, Walter-Huelse-Str. 1, Halle 06120, Germany; E-Mail: frank.altmann@iwmh.fraunhofer.de

**Keywords:** 3D integration, microbump, multiscale microstructure, reliability

## Abstract

The dimensions of microbumps in three-dimensional integration reach microscopic scales and thus necessitate a study of the multiscale microstructures in microbumps. Here, we present simulated mesoscale and atomic-scale microstructures of microbumps using phase field and phase field crystal models. Coupled microstructure, mechanical stress, and electromigration modeling was performed to highlight the microstructural effects on the reliability of microbumps. The results suggest that the size and geometry of microbumps can influence both the mesoscale and atomic-scale microstructural formation during solidification. An external stress imposed on the microbump can cause ordered phase growth along the boundaries of the microbump. Mesoscale microstructures formed in the microbumps from solidification, solid state phase separation, and coarsening processes suggest that the microstructures in smaller microbumps are more heterogeneous. Due to the differences in microstructures, the von Mises stress distributions in microbumps of different sizes and geometries vary. In addition, a combined effect resulting from the connectivity of the phase morphology and the amount of interface present in the mesoscale microstructure can influence the electromigration reliability of microbumps.

## 1. Introduction

Three-dimensional (3D) integration is an emerging technology in which multiple materials, technologies, and functional components are vertically stacked and interconnected to form highly integrated micro-nano systems [1]. Three dimensional integration technology has the potential to increase system performance in many applications, from mobile to high-performance computing and high density memories. Techniques such as through-silicon-via (TSV) and fine-pitch bumping allow for a dense integration of heterogeneous technologies. As complementary metal oxide semiconductor (CMOS) technology barely provides the expected performance improvement, this dense integration of heterogeneous technologies is critical in maintaining Moore’s momentum [2,3,4,5,6,7].

Due to technical challenges for stacking up wafers with TSVs for electrical feed through without any bumps, 3D integration with microbumps and thin chips has been favored since 2000, e.g., memoery-chips stacking, active interposers, and passive interposers [5]. Interconnects distribute the clock and other signals, provide ground, and supply power for various components of an electronic system [8]. The ever-growing need for denser integration or more input/outputs (I/Os) requires a scaling down of the dimensions and pitch of microbumps. Scaling the microbump pitch from hundreds to a few tens of microns is not straightforward because ultrafine microbumps require a stricter control of the electrical, thermal, and mechanical properties [9,10,11,12,13]. Ensuring the required reliability of the interconnect is thus critical [2]. Electromigration reliability requires improved interface control; dielectric reliability depends on the barrier-/low-k and chemical mechanical polishing (CMP)-interface control. Stress-induced voiding is driven by stress gradients, and chip-package interactions are increasingly difficult to minimize when porous dielectrics with a low Young’s modulus are used [2].

An interconnect’s lifetime is approximately proportional to the product of the line width and line height. When the dimensions are reduced, the reliability margin also decreases [2]; therefore, to ensure reliable 3D integrations, investigations are required into the microstructure of ultrafine interconnects. Unfortunately, there is a significant shortage of systematic studies on the microstructural formation and consequent microstructure-reliability relationship of ultrafine interconnects in the existing literature. The microstructure of microbumps, which can consist of Sn-based solder and Cu, Au, or In and Au, changes and for some cases intermetallic compounds (IMCs), e.g., Cu_6_Sn_5_, can essentially occupy the complete bump volume, e.g., through solid-liquid interdiffusion bonding (SLID) [14,15]. Intermetallic bumps have a higher melting temperature, thus making them a preferred candidate for 3D stacking without the danger of re-melting the previously made interconnections. It has been reported that SLID bonding provides a superior reliability performance [15]. In terms of growth kinetics, the activation energy for growing IMCs has been extensively reported in the literature (e.g., [16]). However, the material composition and processing can play a role in diffusion and IMC growth kinetics. Knowing only the activation energy and pre-exponential factor for the growth of IMCs is not sufficient for ensuring the reliability of microbumps in 3D integration [17]. For microbumps with dimensions of less than 10 μm, critical data, regarding the process and mechanism of the multiscale microstructural formation, the morphology and size of the phases, and the microstructural effects on reliability, need to be collected and understood to assist in a data-driven design for reliability.

This study aims to systematically investigate multiscale microstructural formation and subsequent microstructural effects on the reliability of microbumps for 3D integration using a modeling and simulation approach. Emphases are placed on the size and geometry effects on the microstructural formation and the relationship between microstructure and reliability of the microbumps. In particular, hourglass-shaped microbumps, which can be formed through a special fabrication process [18], are considered since it has been reported that an hourglass shape could improve joint fatigue lifetime [19]. The structure of the paper is as follows. Section 2 introduces the modeling methodology, including the phase field and phase field crystal models for solidification, the coupling between microstructural evolution and mechanical modeling, and the microstructure-based electromigration modeling. Section 3 is the main body of the paper; Section 3.1 presents the mesoscale microstructural formation during solidification of the microbumps using a phase field method. Atomic-scale microstructural formation during solidification of ultrafine microbumps using a phase field crystal method is presented in Section 3.2. Section 3.3 and Section 3.4 present the microstructural effects on the mechanical and electrical reliability of the microbumps, with the stress effects on the phase separation and coarsening process in Section 3.3 and the coupling of microstructure, elastic mechanics, and electromigration in Section 3.4. Section 4 draws conclusions based on the modeling and simulation results.

## 2. Modeling Methodology

### 2.1. Phase Field Model of Solidification

A phase field model introduced by Kim *et al*. [20] is used to simulate the microstructural evolution during solidification within one half of the cross-section of a microbump. The governing equations of the order parameter, *ϕ*, and the temperature field, *T*, are:

(1) $$\frac{\partial T}{\partial t}=D\nabla^{2}T+30\phi^{2}\left(1-\phi^{2}\right)\frac{\Delta H}{C_{p}}\frac{\partial \phi }{\partial t}$$

(2) $$\frac{\partial \phi }{\partial t}=L\left[\begin{array}{l} \nabla \cdot \left(\varepsilon^{2}\nabla \phi \right)+\frac{\partial }{\partial y}\left(\varepsilon \frac{d\varepsilon }{d\theta }\frac{\partial \phi }{\partial x}\right)-\frac{\partial }{\partial x}\left(\varepsilon \frac{d\varepsilon }{d\theta }\frac{\partial \phi }{\partial y}\right)\\ -2w\phi \left(1-\phi \right)\left(1-2\phi \right)-30\phi^{2}{\left(1-\phi \right)}^{2}\frac{\Delta H}{T_{m}}\left(T-T_{m}\right) \end{array}\right]$$

where *D* is the thermal diffusivity; ∆*H* is the latent heat per unit volume; *C_p_* is the heat capacity per unit volume; *L* is the phase field mobility; ε is the gradient energy coefficient; θ is the angle between the normal vector of the solid-liquid interface and the *x*-axis; *w* is the height of the double-well potential; and *T_m_* is the melting temperature. The gradient energy coefficient, ε is assumed to be six-fold anisotropic [20]:

(3) $$\varepsilon \left(\theta \right)=\varepsilon_{0}\left[1+\delta \cos6\left(\theta -\theta_{0}\right)\right]$$

where δ is the anisotropy and θ_0_ is the orientation corresponding to the maximum anisotropy. In this study, θ_0_ is set to 60°, and the six directions, which have angles of 30°, 90°, 150°, 210°, 270°, and 330° with respect to the *x*-axis, are called the anisotropic directions. The thermal diffusivity of the system is set as:

(4) $$D=\phi D_{S}+\left(1-\phi \right)D_{L}$$

where *D_S_* = 1.55 × 10^−5^ m^2^/s and *D_L_* = 5 × 10^−6^ m^2^/s are the thermal diffusivities of the solid and liquid phases, respectively. Note that this system is different from the model of Kim *et al*. [20], in which the thermal diffusivities are the same in both phases. The other parameters are based on the previous work by Kim *et al*. [20].

The boundary conditions (BCs) for the phase field model are set as follows. Zero-flux BCs are set at all of the boundaries for the phase field. For the temperature field, the curved boundaries are convectional with a coefficient of 2000 W·m^−2^·K^−^^1^. The other boundaries are adiabatic. It is noted that this phase field model was originally used to simulate the solidification process of an undercooled pure material, and as such, it is a comparatively crude model for simulating the microstructure of microbumps. Despite the simplified nature of the simulated microstructure, the general trends of the size and geometry effects on the microstructural formation are expected to provide insight for the study of more realistic systems in which more complex microstructures can form.

### 2.2. Phase Field Crystal Model of Solidification

A phase field crystal (PFC) model introduced by Elder *et al*. [21,22] is used to simulate the solidification process in ultrafine interconnects. The governing equation of the PFC model is:

(5) $$\frac{\partial \psi }{\partial t}=\nabla^{2}\left[\left(r+{\left(1+\nabla^{2}\right)}^{2}\right)\psi +\psi^{3}\right]+\xi$$

where ψ is the time-averaged atomistic density, *r* = −0.25 is the undercooling, and ξ is the stochastic noise, which is not considered in this study. The solidification occurs from the nuclei, whose densities are set as:

(6) $$\psi =A\left[\cos\left(qx\right)\cos\left(qy/\sqrt{3}\right)+\cos\left(2qy/\sqrt{3}/2\right)\right]+\bar{\psi }$$

where *A* = −0.47034, *q* = $\sqrt{3}/2$, and $\bar{\psi }=0.285$. The nuclei are placed at different locations in the interconnects so that the effect of the nucleus location on the atomic arrangements can be studied. In the two-dimensional PFC model, the solid phase is assumed to have a hexagonal lattice, and the density of the liquid phase is set as $\bar{\psi }$. The variables in the PFC model used in this study are dimensionless.

### 2.3. Coupling Microstructural Evolution with Elastic Mechanics

The model of Müller *et al*. [23] is used to simulate the phase separation and coarsening process in binary Sn-Pb alloys. This model incorporates strain energy into the phase separation process as a driving force for microstructural evolution. Therefore, the model can provide more accurate predictions regarding the microstructure of microbumps subjected to mechanical loads. The mass fraction of Sn, denoted as *c*, is used as an order parameter to distinguish different phases in the microstructure. The microstructural evolution is simulated by numerically solving the following equations (note that Einstein’s summation convention is used):

(7) $$\rho_{0}\frac{\partial c}{\partial t}+\frac{\partial J_{i}}{\partial x_{i}}=0$$

(8) $$J_{i}=-\rho_{0}M_{ij}\frac{\partial }{\partial x_{j}}\left(\frac{\partial \varphi }{\partial c}-a_{kl}\frac{\partial^{2}c}{\partial x_{k}\partial x_{l}}+\frac{\partial W_{s}}{\partial c}\right)$$

where ρ_0_ is the mass density of the Sn-Pb alloy; *J_i_* is the diffusion flux; *M_ij_* is the mobility matrix; φ is the minimum between the Gibbs free energy of the α (Pb-rich) phase and the β (Sn-rich) phase; *a_kl_* is a matrix containing quantities related to surface tension; and *W_s_* is the elastic strain energy defined by:

(9) $$W_{s}=\frac{1}{2}\left(\varepsilon_{kl}-\varepsilon_{kl}^{*}\right)C_{klrs}\left(\varepsilon_{rs}-\varepsilon_{rs}^{*}\right)$$

where ε*_kl_* is the total strain tensor; $\varepsilon_{kl}^{*}$ is the thermal strain tensor; and *C_kl__rs_* is the stiffness tensor. It should be noted that all of the material properties mentioned above, *i.e.*, ρ_0_, *M_ij_*, *a_kl_*, and *C_klrs_*, are obtained through experimental data except for *C_klrs_* of the β phase. The *C_klrs_* value of the β phase is set to 1/10 of its true value in this study to clearly demonstrate the influence of microstructure on the mechanical behavior of the microbumps. The stress reaches equilibrium much faster than the microstructure evolves; thus, the following static equation is used to solve for stress at each time step in the simulation:

(10) $$\frac{\partial \sigma_{ij}}{\partial x_{i}}=0$$

The above model is solved using the finite element method (FEM) in this study. The FEM enables a simulation of the phase separation process in microbumps of arbitrary geometries, thus incorporating the geometry effects on microstructural evolution. The geometries of the solder joints reported by Liu *et al*. [19,24], *i.e.*, the barrel and hourglass shapes, are investigated. For the mechanical equations, *i.e.*, Equations (9) and (10), the bottom boundaries are fixed, and the top boundaries are sheared by 0.01 μm to simulate the thermal stress caused by a mismatch in the coefficients of thermal expansion between the substrate and chip. For the microstructural evolution equations, *i.e.*, Equations (7) and (8), a zero-flux condition for *c* is set to all boundaries. Initially, the microbumps are free of stress, and a fluctuation in *c* is imposed at the center of the domain. Microbumps with pad sizes of 1.5, 3, 4.5 and 8 μm in diameter are systematically investigated. The standoff height of each microbump is set to be equal to the pad size. The phase separation and coarsening process is simulated for 80 s, when the phase separation is complete for most of the microbumps studied. During the simulation, the shear modulus of the microbump is calculated according to the following equation:

(11) $$G=\frac{\bar{\tau }}{\Delta x/h}$$

where $\bar{\tau }$ is the average shear stress across the top boundary of the microbump; ∆*x* is shear displacement and set to be 0.01 μm; and *h* is the standoff height.

### 2.4. Microstructure-Based Electromigration Modeling

The electromigration model developed by Sukharev *et al*. [25] is extended for binary alloys in this study. The model incorporates the electric current and stress gradient as driving forces for vacancy diffusion and assumes that vacancy generation and annihilation occur at interfaces between different phases. The governing equation of vacancy diffusion is:

(12) $$\frac{\partial C_{v}}{\partial t}+\nabla \cdot \left(J_{ch}+J_{str}+J_{em}\right)=G$$

where *C_v_* is the mole fraction of vacancy; **J***_ch_*, **J***_str_*, and **J***_em_* are the vacancy flux driven by a gradient of the chemical potential, stress, and electric potential, respectively; and *G* is a term related to the generation/annihilation kinetics of the vacancy.

Vacancy diffusion in the microbumps is a complicated process because it involves diffusion in multi-component systems. However, for simplicity in this study, the following effective vacancy fluxes based on the derivation of Darken’s equation for interdiffusion [26] are used:

(13) $$J_{ch}=-D_{v}\nabla C_{v}-\frac{D_{v}C_{v}}{kT}\nabla E^{f}$$

(14) $$J_{str}=-\frac{D_{v}C_{v}}{kT}\nabla \left(f\Omega \sigma_{h}\right)$$

(15) $$J_{em}=\frac{C_{v}e\nabla V}{kT}\left(C_{A}D_{A}Z_{A}+C_{B}D_{B}Z_{B}\right)$$

where *k* is Boltzmann’s constant; *T* is the absolute temperature; *E^f^* is the vacancy formation energy; Ω is the atomic volume; *f* is the ratio between the volume of the vacancy and the atom; σ*_h_* is the hydrostatic stress; *e* is the electron charge; *V* is the electric potential; *C_A_*, *C_B_*, *D_A_*, *D_B_*, *Z_A_* and *Z_B_* are the mole fractions, intrinsic diffusion coefficients, and effective charge numbers of components A and B in an A-B binary alloy, respectively; and *D_v_* is the interdiffusion coefficient expressed as:

(16) $$D_{v}=C_{B}D_{A}+C_{A}D_{B}$$

The diffusion coefficient at the interface is set as 100 times that of the bulk phase to simulate short-circuit diffusion.

The generation/annihilation term in Equation (12) is [25]:

(17) $$G=-\frac{C_{v}-C_{v}^{eq}}{\text{τ}}I$$

where:

(18) $$C_{v}^{eq}=\exp\left(\frac{-E^{f}+\left(1-f\right)\Omega \sigma_{h}}{kT}\right)$$

is the mole fraction of vacancy in the thermodynamic equilibrium state in a microbump subjected to stress. This mole fraction is dependent on the vacancy formation energy, *E^f^*, and the hydrostatic stress, σ*_h_*; τ is the time related to the rate of vacancy generation and annihilation.

To predict a more accurate stress distribution at the interface, the atom exchange between the bulk phase and the interface is considered by introducing the concept of “a plated atom” [25]. It is assumed that plated atoms are generated or annihilated simultaneously with the vacancies at the interfaces and that the plated atoms cannot move due to their relatively small mobility compared to the vacancies. The following equation calculates the mole fraction of plated atom, *C_p_* [25]:

(19) $$\frac{\partial C_{p}}{\partial t}=G$$

Under a current stressing, the mole fractions of vacancy and plated atom change and thus result in a dilatation effect. Such a dilatation effect introduces an inelastic strain [25] defined as:

(20) $$\varepsilon_{ij}^{em}=\frac{1}{3}\left[-\left(1-f\right)\left(C_{v}-C_{v0}\right)+\left(C_{p}-C_{p0}\right)\right]\delta_{ij}$$

where δ*_ij_* is the Kronecker delta and *C*_*v*0_ and *C*_*p*0_ are the mole fractions of vacancy and plated atom before a current is applied, respectively. When an inelastic strain, $\varepsilon_{kl}^{em}$, is generated, stress will develop in the microbumps. The stress reaches equilibrium much faster than the vacancy diffusion; thus, the following static equation is used [23]:

(21) $$\frac{\partial }{\partial x_{j}}\left[C_{ijkl}\left(\varepsilon_{kl}-\varepsilon_{kl}^{em}\right)\right]=0$$

where ε*_kl_* is the total strain. In this way, the vacancy diffusion and stress evolution, *i.e.*, Equations (12)–(21), are coupled with the following continuity equation for a steady state current [25]:

(22) $$\nabla \cdot \left(\frac{1}{\rho }\nabla V\right)=0$$

where ρ is the electrical resistivity.

The aforementioned model is used to simulate the electromigration process in SnPb and SnCu microbumps at 150 °C for 500 h. In the simulations, the top and bottom boundaries of the joints are fixed, whereas the left and right boundaries can deform freely. The net flux of the vacancies, *i.e.*, **J***_ch_* + **J***_str_* + **J***_em_*, is assumed to be zero at all boundaries of the microbumps. An electric potential of +0.01 V is applied to the bottom boundaries of the microbumps, and the top boundaries are grounded. The initial conditions are set as $C_{v0}=C_{v}^{eq}$ and *C_p_*_0_ = 0. The material properties used in the simulations are listed in Table 1, where *C*_11_, *C*_12_, and *C*_44_ are the components of the stiffness tensor in the Voigt notation, and *D* is the intrinsic diffusion coefficient. Note that Sn exhibits a tetragonal crystal structure at 150 °C, and the material properties of the (110) crystal plane are used. In addition, the effective charge numbers of Sn and Pb in the Sn-rich and Pb-rich phases are assumed to be the same as those in pure Sn and Pb, respectively.

**Table 1:** Material properties used in the electromigration model.

Alloy	Phase	*E^f^* (eV)	ρ (µΩ cm)	*C*_11_ (GPa)	*C*_12_ (GPa)	*C*_44_ (GPa)	Diffusion element	*D* (×10^−13^ m^2^/s)	*Z*
SnPb	Sn-rich	0.54 [27]	14.8 [28]	75.29 [23]	61.56 [23]	21.93 [23]	Sn	6.41 [29]	−17 [30]
							Pb	6.41 [29]	−47 [30]
	Pb-rich	0.50 [27]	30.3 [28]	49.66 [23]	42.31 [23]	14.98 [23]	Sn	2.15 [29]	−17 [30]
							Pb	5.33 [29]	−47 [30]
SnCu	Sn-rich	0.54 [27]	14.8 [28]	75.29 [23]	61.56 [23]	21.93 [23]	Sn	6.41 [29]	−17 [30]
							Cu	201 [31]	−2 [32]
	Cu_6_Sn_5_	1.28 [27]	17.5 [33]	164.95 [31]	74.11 [31]	45.42 [31]	Sn	6.49 × 10^−3^ [32]	−36 [32]
							Cu	7.04 × 10^−3^ [32]	−26 [32]

## 3. Results and Discussion

### 3.1. Mesoscale Microstructural Formation during Solidification

This section discusses the mesoscale microstructural formation during the solidification of microbumps with an emphasis on the size and geometry effects on the microstructural formation. Note that a phase field model with a multi-component and multi-phase nature is beyond the scope of this study. Therefore, the solidification model used by Kim *et al*. [20] for pure materials is adopted. Despite this simplification, the features of microstructural formation during solidification and the size and geometry effects on microstructural formation in microbumps can be studied.

#### 3.1.1. Size Effects on Microstructural Formation

**Figure 1:**
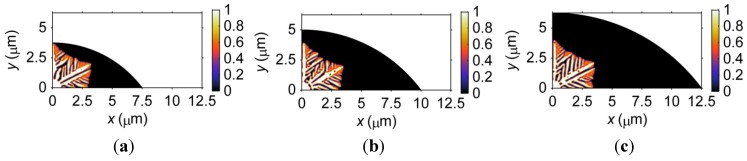
Microstructure of three barrel-shaped microbumps (only half is shown in the figure, and the symmetric line is the *y*-axis) with different sizes at *t* = 5 × 10^−8^ s after solidification. Note that the ratio between the standoff height and the pad size of the microbump is kept the same, *i.e.*, 1:4, as the size of the microbump changes. (**a**) 3.75-μm standoff height; (**b**) 5-μm standoff height; (**c**) 6.25-μm standoff height.

Figure 1 demonstrates the microstructures of three microbumps with different sizes at *t* = 5 × 10^−8^ s after solidification. Note that the ratios between the standoff height and the pad size (only half is shown in Figure 1) of the three microbumps are kept the same, *i.e.*, 1:4. A nucleus is assumed to form at the bottom left corner of the microbump as an initial condition for solidification. Differences in the morphology of the dendrites in the three microbumps can be observed. In the 3.75-μm-standoff height microbump, the 90° primary dendritic arm (hereinafter referred to as the 90° arm) has already reached the top boundary, and the 30° primary dendritic arm (hereinafter referred to as the 30° arm) splits into two branches. In the 5-μm-standoff height microbump, the 90° arm splits into two branches. Note that only one branch of the 90° arm is shown in Figure 1b; the other branch is symmetric to that shown along the *y*-axis. The 30° arm also splits into two branches, but the branches exhibit obvious differences in both size and morphology, *i.e.*, one branch dominates the other, showing a relatively fully developed dendritic structure. In the 6.25-μm-standoff height microbump, both the 90° and 30° arms split. This phenomenon of the dendritic arm splitting has been reported by other researchers (e.g., [34]) and is believed to be caused by numerical errors. In addition, the morphologies of the secondary dendritic arms between the 30° and 90° arms are slightly different among the three microbumps. Overall, there is no major difference in microstructure for the three microbumps studied here. However, the results indicate that a minute numerical error can propagate and thus influence the microstructural formation.

**Figure 2:**
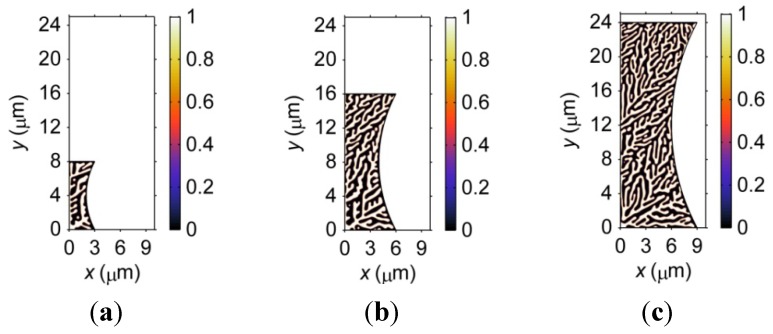
Microstructure of three hourglass-shaped microbumps with different standoff heights at *t* = 5 × 10^−7^ s after solidification. Note that the ratio between the standoff height and the pad size of the microbumps is kept the same as the standoff height of the microbump changes. (**a**) 8-μm standoff height; (**b**) 16-μm standoff height; (**c**) 24-μm standoff height.

To further investigate the size effect on the microstructural formation during solidification, Figure 2 shows the microstructure at *t* = 5 × 10^−7^ s after solidification in three hourglass-shaped microbumps with substantial differences in standoff heights, *i.e.*, 8, 16 and 24 μm. The effect of size on the microstructure of the microbumps can be clearly observed in Figure 2. It is obvious that a larger microbump contains longer primary dendritic arms. In addition, it is noted that the degree to which the 30° arms deviate from the 30° direction increases as the standoff height increases. This phenomenon can be understood as follows. As a larger microbump contains more dendritic arms, the competition among the dendritic arms is more intense than in a smaller microbump, resulting in the deviation of the 30° arm from its ideal direction. Furthermore, it can be observed from Figure 2 that, in general, the width of the dendritic arm decreases as the standoff height increases. In other words, a larger microbump contains finer dendrites. Moreover, the microstructure of a larger microbump, due to its larger dimensions, appears to be more homogeneous.

The size effects on the microstructure of microbumps with different standoff heights, as discussed above, raise the question of why such size effects are not observed in Figure 1. Considering the microstructural formation process of microbumps of different sizes can shed some light onto this problem. The formation of dendrites goes through three stages: nucleation, growth, and coarsening. In this study, a solid nucleus is placed at the bottom left corner of the microbump as an initial condition for solidification; therefore, the nucleation process is ignored. The time span required for the growing dendrites to touch the boundaries of the bump is less in a smaller microbump. When dendrites fully occupy a microbump, the coarsening stage begins. As a result, the dendrites in a smaller microbump enter the coarsening stage earlier and, thus, exhibit a coarser microstructure. Therefore, the reasons that the size effects are not obvious in Figure 1 is two-fold. First, the difference in size of the three microbumps is small. Second, not enough time is allowed for solidification, and the formation of dendrites has not reached the coarsening stage.

#### 3.1.2. Geometry Effects on Microstructural Formation

**Figure 3:**
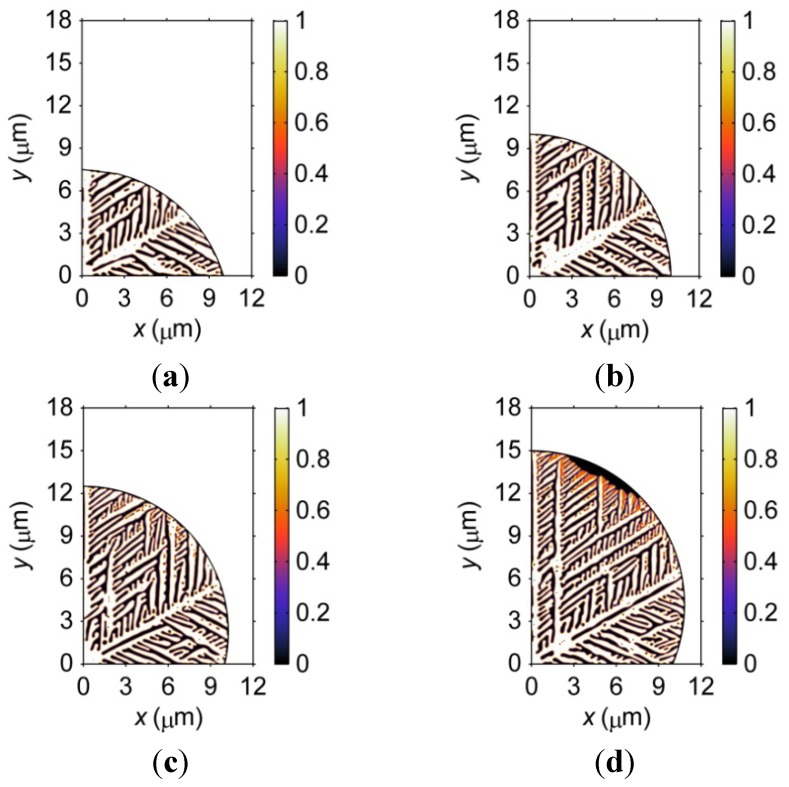
Microstructure of four barrel-shaped microbumps with different geometries at *t* = 2 × 10^−7^ s after solidification. Note that the pad sizes of the microbumps are kept at 20 μm. (**a**) 7.5-μm-height microbump; (**b**) 10-μm-height microbump; (**c**) 12.5-μm-height microbump; (**d**) 15-μm-height microbump.

Figure 3 demonstrates the microstructure of four barrel-shaped microbumps with different geometries at *t* = 2 × 10^−7^ s after solidification. Note that that the pad sizes are all set as 20 μm, but the standoff heights are 7.5, 10, 12.5 and 15 μm. In this case, when the geometry of the microbump changes, the standoff height varies. Therefore, the influence on microstructural formation comes from a combined effect of both the size and geometry. Differences in the morphology of the dendrites can be observed in the four microbumps shown in Figure 3. Apart from the 15-μm-standoff height microbump, the dendrites fully occupy the microbump. As discussed in the previous section on size effects, a larger microbump contains longer primary and secondary dendritic arms. The dendritic structures in the region between the 30° and 90° arms are different in the four microbumps. The fact that a larger microbump contains a finer dendritic structure can also be observed in Figure 3.

The geometry influences the microstructural formation through the curved boundary. In the solidification model, heat from the microbump is exchanged with the environment through the curved boundary by convection, with a convection coefficient of 2000 W·m^−2^·K^−^^1^. However, the cooling effect from the curved boundary is not obvious in Figure 3 because the dendritic structure does not obviously change near the curved boundary.

**Figure 4:**
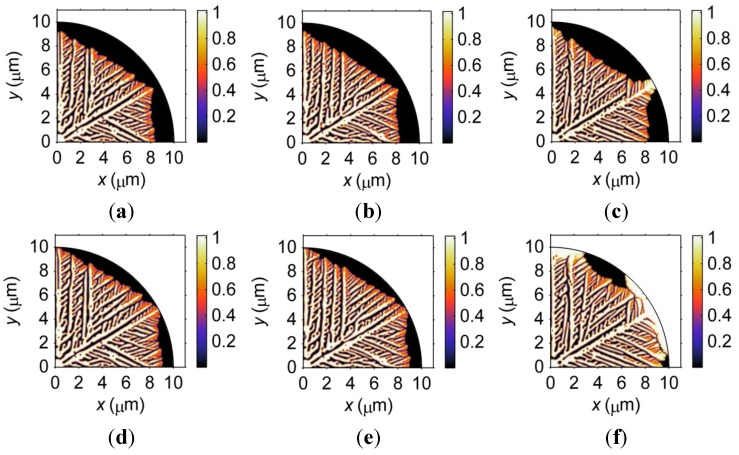
Microstructure of a microbump during solidification at (**a**–**c**) *t* = 1 × 10^7^ s; and (**d**–**f**) *t* = 1.1 × 10^7^ s when the convection coefficient at the curved boundary is set to (a,d) 0 W·m^−2^·K^−^^1^; (b,e) 1000 W·m^−2^·K^−^^1^; and (c,f) 1 × 10^7^ W·m^−2^·K^−^^1^.

To investigate this point further, Figure 4 demonstrates the resulting solidification microstructures when different convection coefficients are set for the curved boundary. Note that the cooling effect from the curved boundary occurs, as shown in Figures 4c,f, when a convection coefficient of 1 × 10^7^ W·m^−2^·K^−^^1^ is used. In reality, the natural convection coefficient in air is much less than 2000 W·m^−2^·K^−^^1^, explaining why the geometry effect is not obvious in Figure 4a,b,d,e. It is worth noting that the simulated dendritic microstructure is a simplified version of the real microstructure of microbumps. First, mass diffusion is not considered in the solidification model. It is expected that the curved boundary will influence the diffusion kinetics and, hence, the solidified microstructure of real microbumps. Second, an undercooling of 200 K is used in the model to accelerate the solidification process; therefore, the heat generated during solidification cannot leave through the curved boundary unless a high heat exchange coefficient is set. As such, the geometry effects on the microstructural formation of real microbumps may be more complicated.

### 3.2. Atomic-Scale Microstructural Formation during Solidification

#### 3.2.1. Solidification Rate

**Figure 5:**
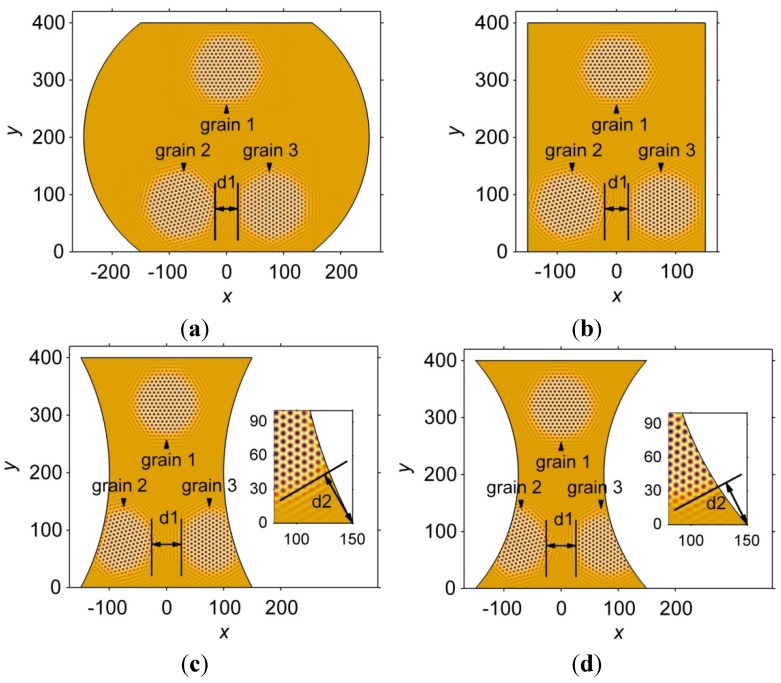
Atomic-scale microstructure in interconnects with (**a**) barrel; (**b**) rectangle; (**c**) hourglass; and (**d**) thinner hourglass geometries at a non-dimensional time, *t* = 150. The inserts in (c,d) show the atomic arrangements at the bottom right corners of the interconnects.

Figure 5 demonstrates the atomic-scale microstructure inside the interconnects of different geometries at the same non-dimensional time, *t* = 150. In the simulations, three nuclei are placed at the same positions inside the interconnects, *i.e.*, at the coordinates (−80,80), (80,80) and (0,320). The nuclei solidify to form grains 1–3, as labeled in Figure 5. The three grains are of different orientations, *i.e.*, 0°, 15° and 30°, respectively. The yellow region in Figure 5 represents the undercooled liquid phase. The distance marked “d1” in each interconnect measures the distance between the solidification fronts of grains 2 and 3. The distance marked “d2” in the inserts of Figure 5c,d measures the distance between the bottom right corner, *i.e.*, (0,150), and the solidification front, which is indicated by the solid line, of grain 3. Two inserts are included in Figure 5c,d to more clearly show the atomic arrangement at the bottom right corner. The results indicate that the solidification rates of the grains are influenced by the geometry of the interconnects. The grains in the barrel-shaped and rectangular interconnects grow faster than those in the two hourglass-shaped interconnects, as suggested by the “d1” values. However, there is only a slight difference between the “d1” values in the barrel-shaped and rectangular interconnects because the amounts of undercooled liquid in the interconnects are different due to their geometries. In the barrel-shaped and rectangular interconnects, the amount of undercooled liquid can provide a sufficient supply for the solidification process. The two hourglass-shaped interconnects, on the other hand, contain less undercooled liquid, and the solidification process slows down due to the limited supply. The geometry effect on the solidification rate is more obvious when the grains solidify along the curved boundary. For example, grain 3 in the thinner hourglass-shaped interconnect solidifies more quickly than that in the wider hourglass-shaped interconnect, as indicated by “d2” values shown in the inserts of Figure 5c,d. Note that the curvature of the curved boundary in the thinner hourglass-shaped interconnect is larger, suggesting that the solidification rate of grain 3 increases with the curvature of the boundary.

#### 3.2.2. Grain Boundary Formation

**Figure 6:**
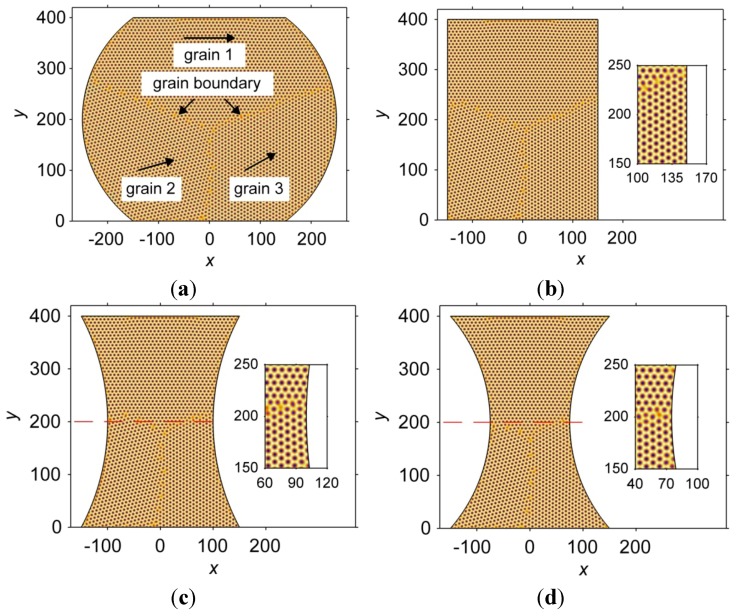
Atomic-scale microstructure in interconnect with (**a**) barrel; (**b**) rectangle; (**c**) hourglass; and (**d**) thinner hourglass geometries at a non-dimensional time, *t* = 1000. The inserts in (b–d) show the atomic arrangements close to the right boundary at 150 < *y* < 250.

Figure 6 demonstrates the atomic-scale microstructure of the interconnects of different geometries at a non-dimensional time, *t* = 1000. Three grains with different orientations are found in each interconnect. The orientations of the grains are shown by solid arrows in Figure 6a. Three grain boundaries (GBs), where dislocations aggregate, are also found in each interconnect. Differences can be found with respect to the GBs in the interconnects. The length of the GBs between grains 1 and 3, as well as the dislocation density, differs among the interconnects. In addition, the GB between grains 1 and 2 in the hourglass-shaped interconnect is shifted compared to that in the thinner hourglass-shaped interconnect, as indicated by the dashed lines in Figure 6c,d. The differences in the morphology of the GBs result from both the misorientation of the neighboring grains and the geometrical constraint of the boundary. With such differences existing, the interconnects may exhibit different behaviors when subjected to the same external physical field. For instance, the elastic-plastic deformation behaviors of the interconnects under the same mechanical load may be different. The geometry of the interconnects can also influence the atomic arrangement; the atoms near the curved boundary of the interconnect are constrained to locate along the boundary instead of retaining the hexagonal lattice. Examples of this phenomenon are shown in the inserts of Figure 6b–d. A detailed discussion on this effect follows.

#### 3.2.3. Atomic Arrangement

**Figure 7:**
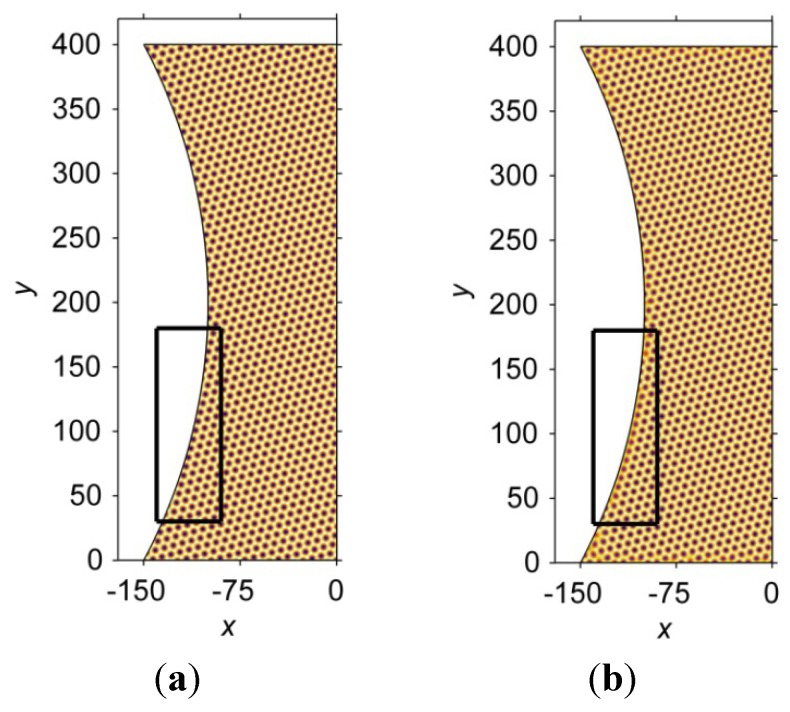
The atomic arrangement in an hourglass-shaped interconnect at a non-dimensional time, *t* = 1200. (**a**) A perfect lattice; (**b**) A simulated atomic arrangement.

Figure 7 shows the atomic arrangements in an hourglass interconnect at a non-dimensional time, *t* = 1200. Figure 7a shows the perfect lattice, which is generated using Equation (6) and then cropped by the hourglass geometry. Figure 7b displays the simulated atomic arrangements obtained by solving the PFC governing equation. In the simulations, only the 15° nucleus is placed at position (−80,80) in the interconnects in order to avoid interactions with other grains. The result from the PFC simulation shows that the atomic arrangement is influenced by the geometry. For example, the atomic arrangements in the black rectangles of Figure 7 differ. The simulated atomic arrangement in the black rectangle of Figure 7b forms a GB-like structure, which is not observed in the perfect lattice, as shown in Figure 7a.

To more clearly demonstrate the differences in the atomic arrangements, stream-line plots of the displacements of the atoms in a PFC simulation relative to the corresponding atoms in a perfect crystal lattice are constructed according to the following procedure. First, the centers of the atoms in the two lattices are identified by an image processing method. Second, for every atom A in the perfect lattice, the corresponding atom B in the simulated lattice is found by a criterion stating that B is the atom closest to A in the simulated lattice. Finally, a vector pointing from A to B is drawn to show the displacement.

**Figure 8:**
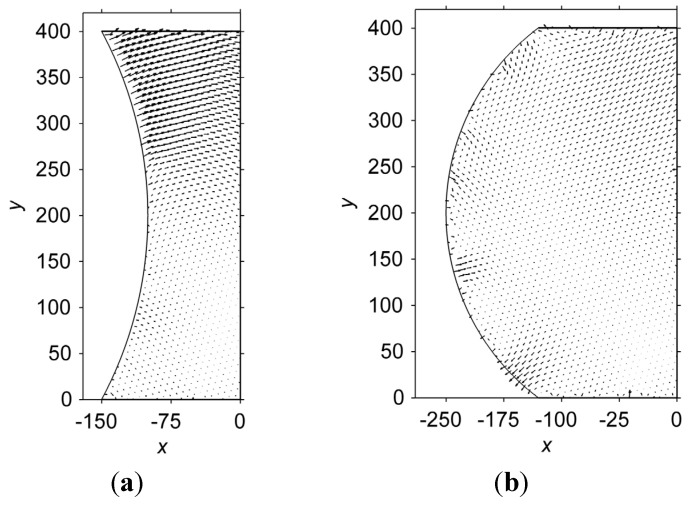
Stream-line plots of the atom displacement in (**a**) the hourglass-shaped; and (**b**) barrel-shaped interconnects. The solid nucleus is placed at (−80,80). Note that the vectors have been magnified by a factor of two, to more clearly show the displacements.

Figure 8 shows stream-line plots of the atom displacement in the hourglass-shaped and barrel-shaped interconnects. The nucleus was placed at the coordinate (−80,80). Note that the vectors are scaled by a factor of two to more clearly show the displacements. In Figure 8a, it is obvious that the atoms above *y* = 250 in the hourglass-shaped interconnect have a relatively larger displacement. In addition, the direction of the displacement rotates clockwise from approximately 15° with respect to the horizontal line at the curve boundary to approximately −15° at the center line of the microbump. For the atoms below *y* = 250, the displacements are virtually zero. In contrast, the displacements of the atoms in the barrel-shaped interconnect exhibit a different behavior, as shown in Figure 8b. The displacements are smaller, and the stream-line plot does not form a global pattern. However, some local patterns do form, e.g., the pattern near the coordinate (−175,25).

**Figure 9:**
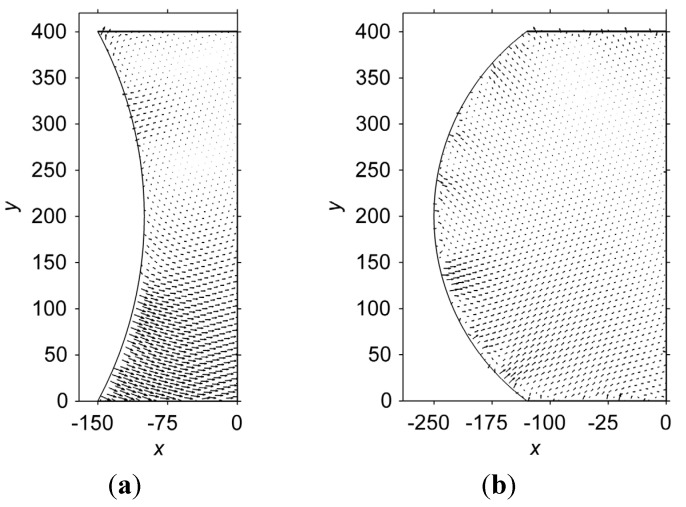
Stream-line plots of the atom displacement in (**a**) the hourglass-shaped and (**b**) barrel-shaped interconnects. The nucleus is placed at (−80,320). Note that the vectors have been magnified by a factor of two, to more clearly show the displacements.

Figure 9 shows stream-line plots for atom displacements in the hourglass-shaped and barrel-shaped interconnects when the nucleus is placed at (−80,320), which is a symmetric point of (−80,80) along the line *y* = 200. In Figure 9a, the steam-line plot of the displacements in the hourglass-shaped interconnect becomes symmetric to the original stream-line plot along the line *y* = 200. For the barrel-shaped interconnect, a change in the nucleus location influences the stream-line plot of the displacement, as shown in Figure 9b. Such changes indicate that the position of the nucleus is a key factor in determining the pattern of the stream-line plot for atom displacement. Furthermore, the displacements in the barrel-shaped interconnect are smaller than those in the hourglass-shaped interconnect, similar to Figure 8. Therefore, the magnitude of the atom displacement depends on the geometry of the interconnect, while the pattern of the displacement distribution is influenced by the location of the nucleus.

### 3.3. Effects of Stress on Solid State Microstructural Evolution

#### 3.3.1. Effects of Stress on Phase Separation

**Figure 10:**
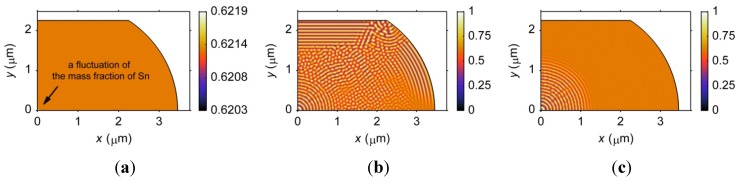
Microstructures formed during the phase separation process in a barrel-shaped Sn37Pb microbump. (**a**) Initial microstructure; (**b**) *t* = 2 s under a shear stress; (**c**) *t* = 2 s free of stress.

The microstructures formed in a barrel-shaped Sn37Pb microbump when under a shear stress and when free of stress are studied to investigate the sensitivity of the microstructure to an external stress. The initial microstructure and the microstructures formed at *t* = 2 s in the phase separation process are demonstrated in Figure 10. Note that only one quarter of the microbump is shown. Figure 10b shows that the phase separation proceeds faster when a shear stress is applied, in particular, at the corner of the microbump, where the stress concentrates. In addition, ordered arrangements of the phases are found when external stress is applied. That is, the phases tend to grow along the boundaries, as shown in Figure 10b.

**Figure 11:**
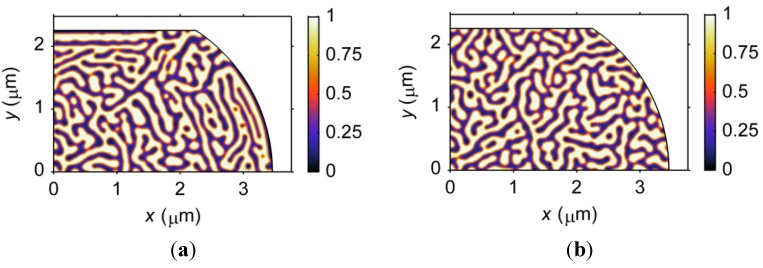
Microstructures formed at *t* = 10 s of the phase separation process in a barrel-shaped Sn37Pb microbump. (**a**) under a shear stress; and (**b**) free of stress. Note that only one quarter of the joints is shown.

This ordered phase arrangement can be observed even if the phase separation is complete, as shown in Figure 11a. In contrast, Figure 11b shows the relatively disordered phase distribution that occurs when no external stress is applied. It should be noted that in Müller’s work, an unrealistic stress level, *i.e.*, 10 GPa, was required to observe a dramatic impact on the microstructural development [23]. However, the maximum stress applied for the result presented in Figure 11 is approximately 300 MPa. The stronger effects of stress on the microstructure in this work are most likely due to the incorporation of geometrical constraints on the diffusion process, which was not considered in Müller’s work. The effects of stress on the microstructure of microbumps should not be neglected. All of the microstructures discussed in the following sections are simulated under a shear stress.

#### 3.3.2. Size Effects on Microstructure

**Figure 12:**
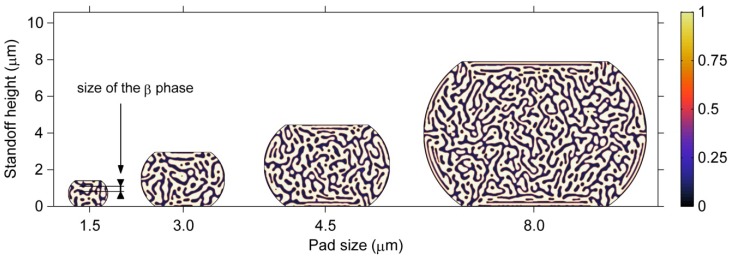
Microstructures of barrel-shaped Sn37Pb microbumps with different pad sizes after the phase separation process has proceeded for 80 s.

The size effect on the phase morphology in ultrafine microbumps is demonstrated in Figure 12. The results show the microstructures after the phase separation process has proceeded for 80 s in barrel-shaped Sn37Pb microbumps of different pad sizes, *i.e.*, 1.5, 3, 4.5 and 8 μm. It is clear that larger phases with respect to the size of the microbump are formed in smaller microbumps. In particular, in the 1.5-μm microbump, the size of the β phase is nearly 1/5 of the standoff height, as shown in Figure 12. Thus, the microstructures of smaller microbumps are highly heterogeneous. In addition, the ordered phase arrangement induced by stress becomes less observable for microbumps with a pad size below 3 μm due to relatively larger phase sizes. A quantitative characterization method must be developed to further study the effects of stress on the microstructure of microbumps at such small scales.

#### 3.3.3. Geometry Effects on Microstructure

**Figure 13:**
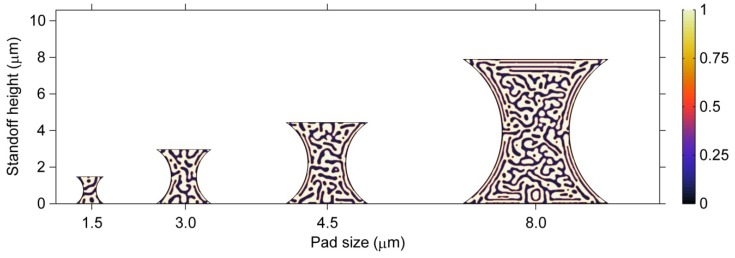
Microstructures of hourglass-shaped Sn37Pb microbumps with different pad sizes after 80 s of the phase separation process.

The effects of geometry on the microstructure of microbumps can be observed by comparing Figure 12 and Figure 13. Figure 13 demonstrates the microstructures after 80 s of the phase separation process for hourglass-shaped Sn37Pb microbumps for the same pad sizes used in Figure 12. A similar relationship between the microstructural heterogeneity and the size of the microbump can be observed for the two geometries. However, the hourglass-shaped microbump contains a larger fraction of the ordered phase region than the barrel-shaped microbump for the same pad size and standoff height. Therefore, the microstructure of an hourglass-shaped microbump exhibits a higher degree of heterogeneity.

**Figure 14:**
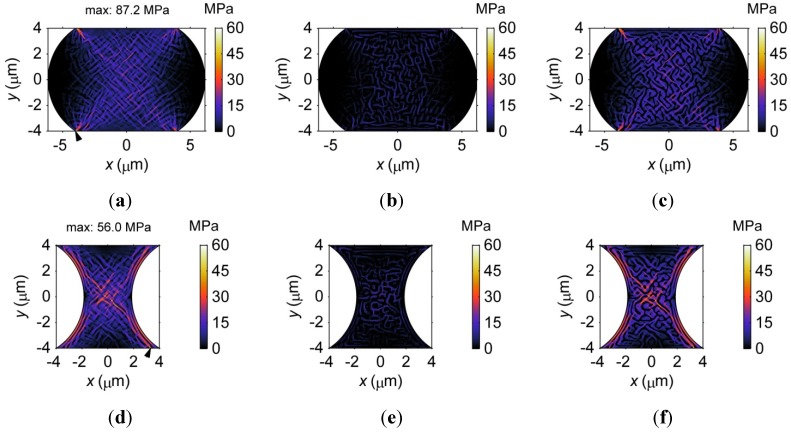
The distribution of von Mises stress in (**a**) a barrel-shaped; and (**d**) an hourglass-shaped Sn37Pb microbump with an 8 μm pad size after 80 s of the phase separation process. (**b**,**c**,**e,f**) show the stress distribution in the α and β phase, respectively. The black triangles in (a,d) indicate the locations of the maximum von Mises stress.

The von Mises stress distributions in the barrel-shaped and hourglass-shaped microbumps with an 8-μm pad size are demonstrated in Figure 14a,d, respectively. To demonstrate the results more clearly, Figure 14b,e display the stress distribution in the α phase, and Figure 14c,f show the stress distributions in the β phase for the barrel-shaped and hourglass-shaped microbumps. It is obvious that stress tends to concentrate in the β phase because the Young’s modulus of the β phase is larger. The maximum stress in the hourglass-shaped microbump is 56 MPa, which is lower than that in the barrel-shaped microbump, *i.e.*, 87.2 MPa, implying that the probability for damage to form and propagate may be lower in an hourglass-shaped microbump. Stress concentrates at different locations in the two geometries. In Figure 14a, higher stress levels are observed at the four corners of the barrel-shaped microbump. In contrast, higher stresses occur near the left and right boundaries and in the center of the hourglass-shaped microbump, as shown in Figure 14d.

Furthermore, stress over 30 MPa only occurs in a small region in the barrel-shaped microbump, as shown in Figure 14a. That is, the microstructure primarily influences the distribution of stress at low levels, *i.e.*, below 20 MPa, in the barrel-shaped microbump. However, the region of stress greater than 30 MPa is larger in the hourglass-shaped microbump, as shown in Figure 14d, where the spatial distribution of stress is primarily determined by the microstructure. The above results suggest that the relationship between the microstructure and von Mises stress in the microbump is also related to the geometry of the microbump.

#### 3.3.4. Shear Moduli of Microbumps

**Figure 15:**
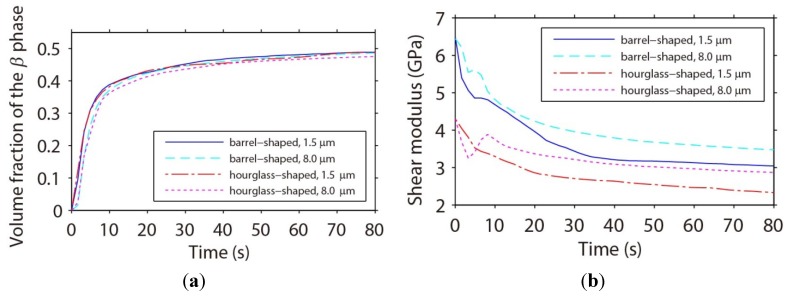
Temporal evolution of (**a**) the volume fractions of the β phase; and (**b**) the shear moduli of the Sn37Pb microbumps of different size and geometries.

During the phase separation and coarsening process, the microstructural evolution can influence the mechanical properties of the microbumps. In particular, the shear moduli of the microbumps change with the microstructural evolution. In this study, the microbumps are under a shear load and the microstructures of the microbumps evolve in order to minimize the strain energy. Figure 15a clearly shows that the volume fraction of the β phase is almost the same for the microbumps of different sizes and geometries. Therefore, the strain energy must be minimized through a change in morphology, *i.e.*, forming the ordered phase structures as shown in Figure 12 and Figure 13. The presence of the ordered phase structures makes the microbumps less stiff under the shear load as indicated by a decrease of the shear modulus as a function of time as shown in Figure 15b. The size and geometry effects on the shear moduli of the microbumps can also be observed from Figure 15b. First, the shear moduli of the microbumps of different sizes and geometries at *t* = 0 s, when microstructure has not yet formed, suggest that the shear moduli of the microbumps are independent of their sizes and determined by their geometries only. The hourglass-shaped microbumps exhibit smaller shear moduli than the barrel-shaped ones throughout the microstructural evolution process from *t* = 0 s to 80 s. Second, the size effect on the shear moduli of microbumps can be observed when microstructure forms and evolves. The shear moduli of the smaller microbumps decrease faster with time. The difference in the shear modulus between the microbumps of the same geometry but with a pad size of 1.5 µm and 8 µm can reach 0.5 GPa at *t* = 80 s.

#### 3.3.5. Composition Effects on Microstructure

**Figure 16:**
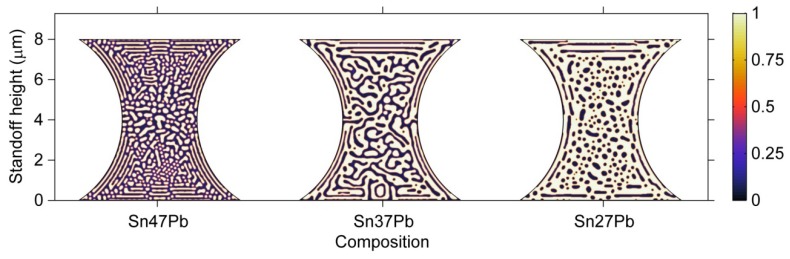
Microstructures of hourglass-shaped microbumps of three different compositions after 80 s of the phase separation process.

The phase separation process in microbumps of three different compositions, *i.e.*, Sn47Pb, Sn37Pb, and Sn27Pb, is studied using an hourglass geometry in this section. According to the above-mentioned results, the interaction between the microstructure and the von Mises stress is stronger in the hourglass geometry. Therefore, the composition effects should be more clearly demonstrated in the hourglass-shaped microbumps. The microstructures of the 8-μm-pad size microbump of the three compositions after 80 s of the phase separation are shown in Figure 16. It can be observed that the volume fraction of the β phase dramatically increases with a decrease in Pb content, which is consistent with the Sn-Pb binary phase diagram; thus, the effect of composition on the microstructure of the microbumps is significant. In the Sn47Pb microbump, the α phase and β phase are separated into small regions, while a completely connected region of β phase is formed in the Sn27Pb microbump. The morphology of the phases also changes with the composition. The β phase in the Sn47Pb microbump is the finest because the mobility of Sn in the Pb-rich regions is lower than that in the Sn-rich regions by three orders of magnitude. Thus, the kinetics of phase separation is relatively slow in the Sn47Pb microbump.

**Figure 17:**
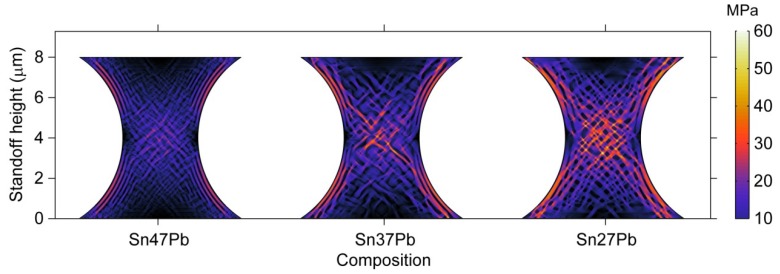
The distribution of von Mises stress in hourglass-shaped solder joints of different compositions after 80 s of the phase separation process.

Figure 17 demonstrates the von Mises stress distribution for the microbumps shown in Figure 16. Because the β phase possesses a higher Young’s modulus, it is expected that an increase in the amount of the β phase would result in a higher stress level in the microbump. In addition, stress tends to concentrate in the β phase, as mentioned earlier. Thus, the distribution of the von Mises stress in the microbump is obviously influenced by the composition.

### 3.4. Effects of Microstructure on Electromigration

#### 3.4.1. Electromigration in Sn27Pb and Sn15Cu Microbumps

**Figure 18:**
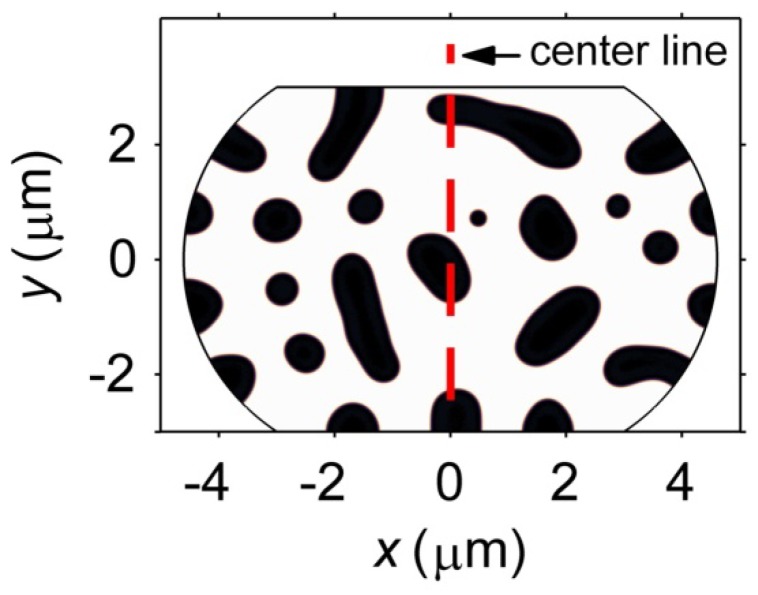
A representative microstructure used in the electromigration simulations. The dashed line is the center line of the microbump.

The vacancy diffusion and stress evolution induced by electromigration in microbumps with compositions of Sn27Pb and Sn15Cu are compared in this section. The effects of microstructure on the distributions of vacancy and stress are also studied. In particular, the differences between the Pb-rich phase and the Cu_6_Sn_5_ IMC phase in determining the electromigration behavior of the microbump are emphasized. The microstructure shown in Figure 18 is used for the electromigration simulations for both the Sn27Pb and Sn15Cu microbumps to eliminate the effects of phase morphology. Further investigations on the effects of morphology on electromigration will be discussed in the following sections. In Figure 18, the light phase represents the Sn-rich phase, whereas the dark phase represents the Pb-rich phase for the Sn27Pb microbump and the Cu_6_Sn_5_ phase for the Sn15Cu microbump.

**Figure 19:**
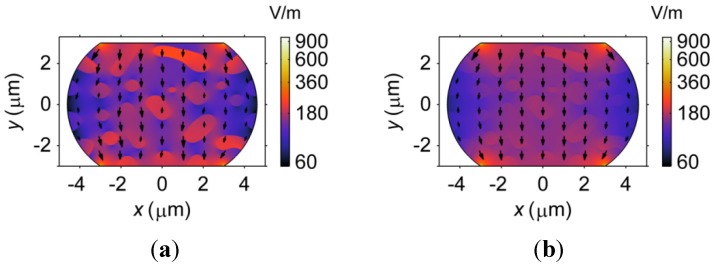
The distribution of the intensity of the electric field, as represented by color shading, in (**a**) Sn27Pb; and (**b**) Sn15Cu microbumps. The arrows in the figure represent the direction and magnitude of the electron flow.

First, the microstructure influences the vacancy diffusion by affecting the distribution of the electric field intensity, as shown in Figure 19. Figure 19 demonstrates that the electric field intensity tends to concentrate at the four corners of the barrel-shaped microbump. However, compared to the Sn-rich phase, a stronger electric field is found in the Pb-rich phase and the Cu_6_Sn_5_ IMC phase due to their higher electrical resistivities. In particular, the intensity of the electric field in the Pb-rich phase is approximately twice as large as that of the Cu_6_Sn_5_ IMC phase, which indicates that the Pb-rich phase provides a larger driving force for the current-induced vacancy flux than the Cu_6_Sn_5_ IMC, according to Equation (15). Second, the direction of the vacancy flux is slightly influenced by the phase morphology, which is indicated by the arrows shown in Figure 19. Such an effect is more obvious in the Sn27Pb microbump, as shown in Figure 19a, because the difference in electrical resistivity between the Sn-rich phase and the Pb-rich phase is approximately six times greater than that between the Sn-rich phase and the Cu_6_Sn_5_ IMC phase.

**Figure 20:**
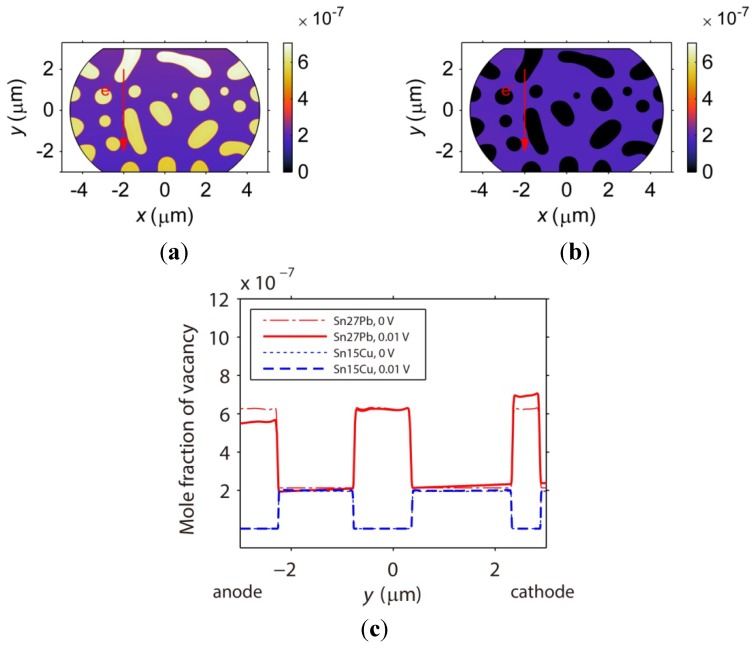
Vacancy distributions of (**a**) Sn27Pb; and (**b**) Sn15Cu microbumps under 0.01 V for 500 h. The vacancy distributions along the center lines of the two microbumps under 0 and 0.01 V are plotted in (**c**).

Other microstructural parameters related to the vacancy diffusion include the vacancy formation energy and parameter *CDZ*, *i.e.*, the product of the mole fraction, diffusivity, and effective charge number, as introduced in Equation (15). First, the vacancies are predicted to accumulate in the phase with the lower vacancy formation energy, *i.e.*, the Pb-rich phase shown in Figure 20a and the Sn-rich phase shown in Figure 20b. Second, the vacancies in the Sn27Pb microbump move more easily under the electric current than those in the Sn15Cu microbump because the *CDZ* in the Pb-rich phase is larger. Figure 20a shows that the vacancies in the Sn27Pb micro joint tend to migrate towards the cathode. In contrast, the mole fraction of vacancy is almost uniformly distributed throughout the phases of the Sn15Cu microbump, as shown in Figure 20b. To clarify this point further, the distributions of the vacancies along the center line shown in Figure 18a before and after current stressing are plotted in Figure 20c. Note that the interfaces in the microstructure studied here are not interconnected. The vacancies must diffuse across the bulk phase to reach the cathode. As a result, fast diffusion of the vacancies along the interfaces does not dominate the electromigration process for the microstructure studied here.

**Figure 21:**
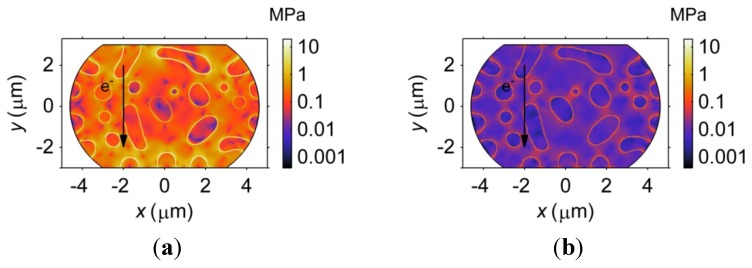
Distributions of von Mises stress in (**a**) Sn27Pb; and (**b**) Sn15Cu microbumps under 0.01 V for 500 h.

**Figure 22:**
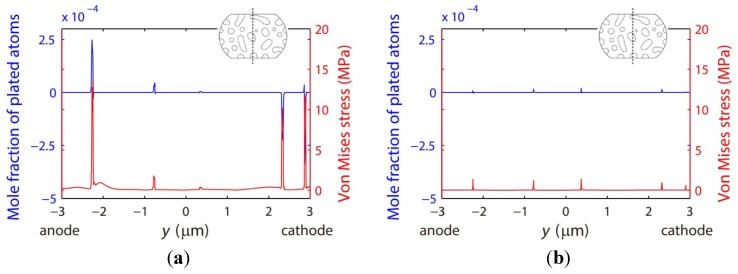
Mole fractions of plated atoms and von Mises stresses along the center lines of (**a**) Sn27Pb; and (**b**) Sn15Cu microbumps after an electromigration process under 0.01 V for 500 h. The inserts show the microstructures and locations of the center lines.

Accompanying the vacancy diffusion process, the internal stress in the microbump evolves due to the dilatation effect. Figure 21 demonstrates that stress in both the Sn27Pb and Sn15Cu microbumps concentrates at the interfaces. As a consequence, the risk of void formation is higher at the interfaces, possibly explaining the experimental observation of voiding at the interfaces in SnPb solder strips [35]. Figure 22 shows the distributions of plated atoms and von Mises stress along the center line shown in Figure 18. It is noted that higher stresses can be found in regions where more plated atoms are generated or annihilated, as shown in Figure 22. Due to this fact, stress concentration is most likely caused by the strain resulting from the generation or annihilation of vacancy-plated atom pairs. In the Sn27Pb microbump, the vacancies are predicted to accumulate at the cathode and deplete at the anode, as shown in Figure 20c. Therefore, more vacancies are annihilated at the cathode and generated at the anode, resulting in higher stress concentrations at both electrodes, as shown in Figure 21a. In contrast, the vacancy distribution in the Sn15Cu microbump remains almost unchanged before and after a current is applied, as shown in Figure 20c. Therefore, the electromigration-induced stress is lower in the Sn15Cu microbump.

#### 3.4.2. Effects of Phase Coarsening on Electromigration

The results discussed above suggest an intimate relationship between the microstructure and electromigration behavior in microbumps. This section further investigates the morphological effects on vacancy diffusion and stress evolution during electromigration. The phase coarsening process in a Sn37Pb microbump is studied first.

**Figure 23:**
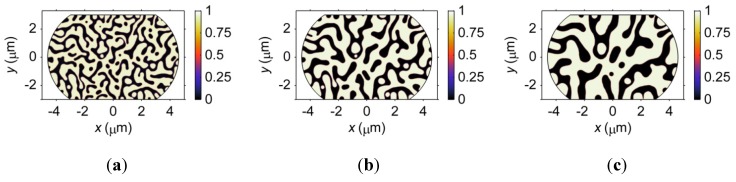
Microstructure of a Sn37Pb microbump aged at 125 °C for (**a**) 300 s; (**b**) 900 s; and (**c**) 3000 s. The color shading represents the mass fraction of Sn.

**Figure 24:**
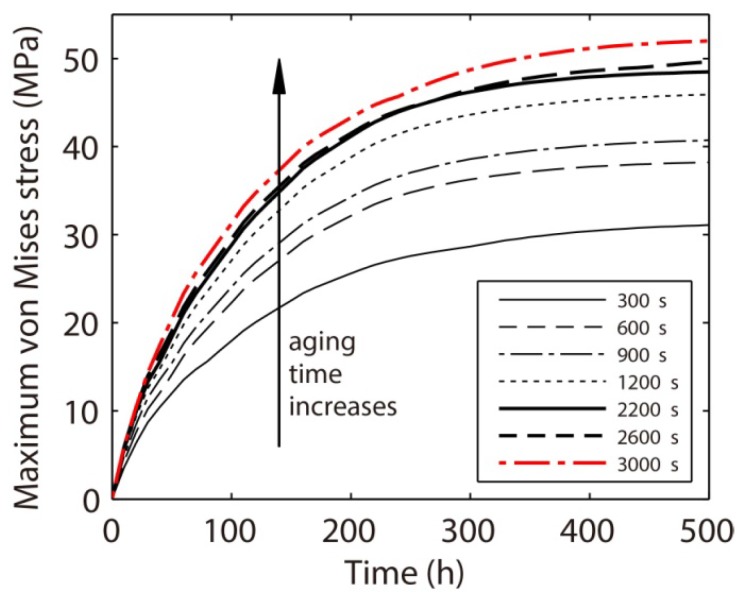
Temporal evolution of the maximum von Mises stress in a Sn37Pb microbump during an electromigration simulation based on a microstructure aged at 125 °C for a specified time, *i.e.*, from 300 to 3000 s.

The microstructures formed in a Sn37Pb microbump after aging at 125 °C for 300, 900 and 3000 s are demonstrated in Figure 23. The volume fractions of the Pb-rich phase are nearly the same for the three microstructures, *i.e.*, 40.09%, 39.93% and 39.81%. The major changes in phase morphology during a coarsening process lie in the size of the phase and the amount of interface. Figure 24 shows the temporal evolution of the maximum von Mises stress in the Sn37Pb microbump during an electromigration simulation based on a microstructure aged at 125 °C for 300 s to 3000 s. The amount of interface is high at the initial stage of the coarsening process, as shown in Figure 23a. At this time, the vacancies can reach vacancy sources or sinks after diffusing a relatively short distance. As a result, vacancy generation and annihilation occur more quickly, mitigating vacancy accumulation and releasing stress concentration. Therefore, the maximum stress in the microbump aged for 300 s is the lowest. After aging for a longer time, the amount of interface decreases, as shown in Figure 22c and Figure 23b. In these cases, the vacancies move longer distances before encountering vacancy sinks, resulting in vacancy accumulation and stress concentration.

**Figure 25:**
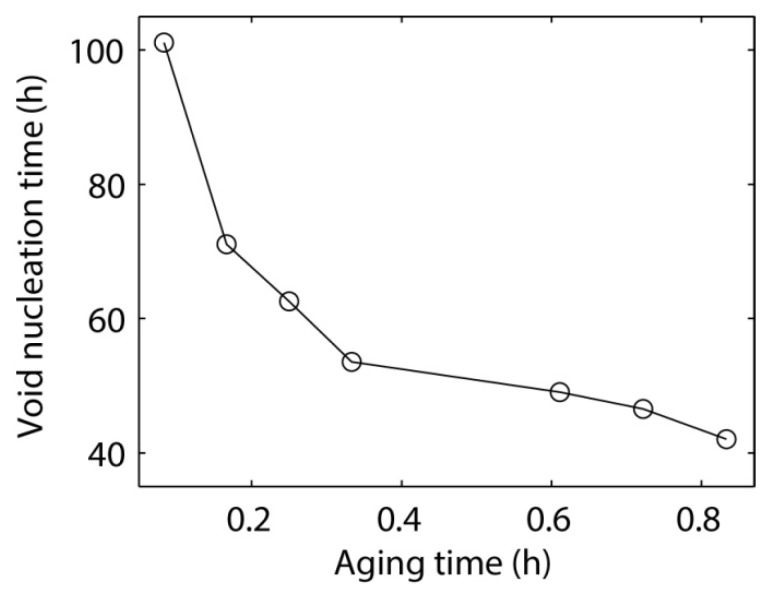
The void nucleation time in a Sn37Pb microbump during the electromigration process as a function of the aging time.

Voiding likely occurs in the microbump when the stress caused by electromigration exceeds the yield stress of the microbump [36]. In this study, the void nucleation time is defined as the time when the von Mises stress reaches the yield stress of the Sn37Pb solder, *i.e.*, 18.1 MPa [37]. The void nucleation time is found to decrease as the aging time increases, as shown in Figure 25; however, the microstructure in the microbump does not remain constant during the electromigration process. The electric current applied to the microbump can also accelerate the phase coarsening process by increasing the system temperature through Joule heating. Therefore, the void nucleation time may be overestimated in this study. Further investigations into the coupling between electromigration and microstructural evolution are currently ongoing to provide more accurate predictions on the lifetime of electromigration.

#### 3.4.3. Effects of Composition on Electromigration

**Figure 26:**
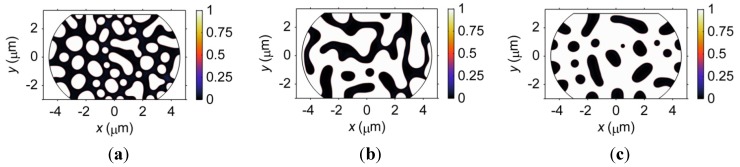
The microstructures of (**a**) Sn47Pb; (**b**) Sn37Pb; and (**c**) Sn27Pb microbumps. The color shading represents the mass fraction of Sn.

The effects of composition on microstructure are more complicated than the phase coarsening case because the amount of each phase and the amount of interface are both related to the composition. The microstructures of microbumps with compositions of Sn47Pb, Sn37Pb, and Sn27Pb are demonstrated in Figure 26. The corresponding volume fractions of the Pb-rich phase are 52.38%, 40.56% and 29.04%, respectively. As shown in Figure 26, the change in the volume fraction of the Pb-rich phase is accompanied by a change in the connectivity of the Pb-rich phase, which can affect the diffusion path for the vacancies. Figure 26a shows that the Pb-rich phase in the Sn47Pb microbump is completely interconnected. An interconnected Pb-rich phase can provide a fast diffusion path for vacancies from the anode to the cathode under an electric current because the vacancy can move more easily in the Pb-rich phase than in the Sn-rich phase. In contrast, the Pb-rich phase in the Sn27Pb microbump is scattered in the Sn-rich matrix, as shown in Figure 26c. In this case, vacancies driven by the electric current from the anode to the cathode must travel through the Sn-rich phase. As a result, vacancy accumulation at the cathode and depletion at the anode are slower in the Sn27Pb microbump. This result also indicates that the maximum stress in the three microbumps should be in the order of Sn47Pb > Sn37Pb > Sn27Pb.

**Figure 27:**
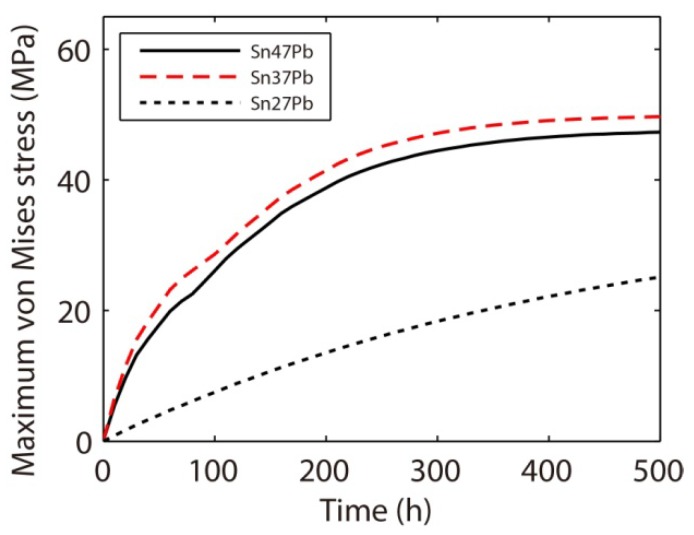
Temporal evolutions of the maximum von Mises stress during electromigration in Sn47Pb, Sn37Pb, and Sn27Pb microbumps.

The opposite conclusion may be drawn if we consider the effect of vacancy generation and annihilation at the interface. That is, a relatively larger amount of interface in the Sn-47Pb microbump, as shown in Figure 26a, can reduce the maximum stress by rapid generation or annihilation of vacancies, as discussed above. Figure 27 shows that the combined effects of the connectivity of the Pb-rich phase and the amount of interface result in a non-monotonic relationship between the maximum von Mises stress and the Pb content. The calculated maximum stresses are in the order of Sn37Pb > Sn47Pb > Sn27Pb, but it should be noted that the maximum stress in the Sn37Pb microbump is only slightly larger than that in the Sn47Pb microbump. This result may explain the experimental observation of rapid hillock formation in eutectic SnPb solder strips [35].

## 4. Conclusions

This paper simulates mesoscale and atomic-scale microstructural formation during solidification and discusses the size and geometry effects on the microstructures of ultrafine microbumps. In addition, simulations on coupled microstructural evolution, mechanical stress, and electromigration have been performed, and the microstructural effects on the reliability of microbumps have been highlighted. The following conclusions can be drawn:

1. The size and geometry of the microbumps can influence the mesoscale microstructural formation during solidification; a smaller microbump solidifies more quickly than a larger counterpart. The length of the dendritic arms increases with the size of the microbump, and a larger microbump contains finer dendrites and a relatively more homogeneous microstructure.
2. The simulated atomic arrangements obtained during solidification using the phase crystal method exhibit a displacement when compared to the ideal crystal lattice due to the combined effects of the location of the nucleus and the geometry of the microbump. The magnitude of the displacement depends on the geometry of the microbump, while the distribution of the displacement is controlled by the location of the nucleus.
3. Ordered phase growth along the boundaries of the microbump can be observed due to influences from the imposed external shear stress and the geometrical constraints on diffusion. The formation of the ordered phase structures reduces the shear moduli of the microbumps to minimize the strain energy. The shear moduli of the hourglass-shaped microbumps are smaller than the barrel-shaped ones and decrease faster in the smaller microbumps as the microstructures evolve with time.
4. More vacancy accumulation and stress concentration occur in the Sn27Pb microbump compared to the Sn15Cu microbump during electromigration. Hence, a lower electromigration reliability of the Sn27Pb microbump can be expected. It is also found that phase coarsening in the Sn37Pb microbump reduces the amount of interface and accelerates vacancy accumulation at the cathode and depletion at the anode. Therefore, stress can quickly increase during electromigration in Sn37Pb microbumps aged for longer times, thus increasing the risk of voiding.
5. A combined effect caused by the connectivity of the Pb-rich phase and the amount of interface should be considered when comparing the electromigration behaviors of the Sn47Pb, Sn37Pb, and Sn27Pb microbumps. The maximum von Mises stress present in the Sn37Pb microbump increases with the electromigration time and depends on the composition of the microbump, which could result in nonuniformity in bump heights if the process variables are not tightly controlled.
